# Supplementary material for: The uptake of WHO-recommended birth preparedness and complication readiness messages during pregnancy and its determinants among Ethiopian women: A multilevel mixed-effect analyses of 2016 demographic health survey
Source: PLoS One. 2023 Mar 23;18(3):e0282792. doi: 10.1371/journal.pone.0282792 (PMC10035894; doi:10.1371/journal.pone.0282792)
Supplement: Attachment — Submitted filename: WHO BPCR Report.docx [file pone.0282792.s001.docx]

**Manuscript title:** The uptake of WHO-recommended Birth Preparedness and complication readiness messages and its determinants in Ethiopia: A multilevel mixed-effects analyses of the 2016 Ethiopian demographic health survey

Comment 1: Table1: Number of BPCR messages received by women during their recent pregnancy

preceding the survey in Ethiopia, 2016.

1. Say something about the table since audiences/Readers did not understand the meaning.

Comment 2: Do not use abbreviation in the abstract part of the document.

Comment 3: Describe EDHS 2016 Collected data under Ethical considerations.

Comment 4: The study relied on population-based, nationally representative data from 2016 Ethiopian

Demographic and Health Survey (DHS), the seventh in a series of national-level population

and health surveys carried out as part of the global Demographic and Health Survey (DHS)

program. **So, why only use EHDS 2016 Data for this study since the title on WHO Recommendations on BPCR messages.**

Comment 5: this paper did not describe about the population characteristics.

Comment 6: Is there any exclusion data? Not described

Comment 7: Did you check Completeness of Data?

Comment 8: under limitation of the study, it says ''retrospective interviews of a selected group of women who had a live birth

within five years of the survey''. Rewrite it.

Comment 9: What were/was drawback of EDHS2016 So far? you did not well explained.

Comment 10: Discussion should be rewrite it again.
