# Supplementary material for: The uptake of WHO-recommended birth preparedness and complication readiness messages during pregnancy and its determinants among Ethiopian women: A multilevel mixed-effect analyses of 2016 demographic health survey
Source: PLoS One. 2023 Mar 23;18(3):e0282792. doi: 10.1371/journal.pone.0282792 (PMC10035894; doi:10.1371/journal.pone.0282792)
Supplement: Attachment — Submitted filename: Response to Reviewers.docx [file pone.0282792.s002.docx]

**AUTHORS’ RESPONSE TO ACADEMIC EDITOR AND REVIEWERS**

1. **Author’s Response To Academic Editor**

**Dear: Melkamu Merid Mengesha, MPH (Academic Editor)**

We thank you for a thorough reading and constructive comments and suggestions on our manuscript and for the opportunity to revise and resubmit. We are pleased to submit the revised research article “**The uptake of WHO-recommended birth preparedness and complication readiness messages during pregnancy and its determinants among Ethiopian women: A multilevel mixed-effect analyses of 2016 demographic health survey**” for your consideration in the special collection of Plos One. On the following pages, you will find our responses to the questions raised from your side and two reviewers' comments. On behalf of my co-authors, I thank you for your consideration of this resubmission. We appreciate your time and look forward to your response.

Sincerely,

Aklilu Habte(MPH)(corresponding author)

[**aklilihabte57@gmail.com**](mailto:aklilihabte57@gmail.com)

**Dear Dr. Habte,**

Thank you for submitting your manuscript to PLOS ONE. After careful consideration, we feel that it has merit but does not fully meet PLOS ONE’s publication criteria as it currently stands. Therefore, we invite you to submit a revised version of the manuscript that addresses the points raised during the review process.

**Response:** Thank you for providing us with the opportunity to revise our work by incorporating all of the valuable comments and suggestions made by the reviewers. We are also grateful for the reviewers' insightful comments and suggestions, which we received all of them were beneficial for further improving the manuscript. In the "Response to Reviewers" section below, we endeavored to answer all constructive remarks and ideas raised by both reviewers.

_____________________________END________________________________________

THANK YOU!!!

**AUTHORS’ RESPONSE TO REVIEWERS**

**Dear: Reviewers**

We thank you for a thorough reading and constructive comments and suggestions on our manuscript and for the opportunity to revise and resubmit. We are pleased to submit the improved research article **“The uptake of WHO-recommended birth preparedness and complication readiness messages during pregnancy and its determinants among Ethiopian women: A multilevel mixed-effect analyses of 2016 demographic health survey”** for your consideration in the special collection of Plos One. On the following pages, you will find our response to the reviewers’ comments. On behalf of my co-authors, I thank you for your consideration of this resubmission. We appreciate your time and look forward to our responses to the comments and suggestions raised from your side independently as “Authors’ response to Reviewer#1”, and “Authors’ response to Reviewer#2”.

Sincerely,

Aklilu H. (MPH)(corresponding author)

[**aklilihabte57@gmail.com**](mailto:aklilihabte57@gmail.com)

**Authors’ Response to Reviewer**#**1**

**General comments**

**Comment 1:** Included samples (women) are not specified whether they are ever pregnant within 5 years preceding a survey or pregnant during the survey.

**Response:** Thank you very much for taking the time to review our work and for your important inquiry. The included samples in this DHS report were women who were pregnant within five years preceding the survey. We have corrected the statement in the "the population of the study" section of the “Revised manuscript with track changes”, Line 134-139, Page 5.

**Comment 2:** In the result (both in the abstract and main result) the result of the random effect is not described.

**Response**: We appreciate your thorough review and allowing us to revise our writeup for multi-level mixed effect analyses in a deserving way. As per your suggestion, we have included reports on the analysis and presentation of random effect statistics in the following sections below:

1. “Model building and selection” section of the revised manuscript“Revised manuscript with track changes”, Line251-259, Page 8 and
2. Again the results of random statistics were presented in Table 5, Page 19, and table 6, page 22 of the “Revised manuscript with track changes”

**Comment 3:** The authors used t-test and ANOVA to associate the frequency of BPCR, however, they suggest a mixed effect model as best fitted data for their data. So, how does data that didn’t violate the assumption for t-test (independence of observation) and ANOVA (independence of observation, equal variance) do violet for multivariate? Did you consider autocorrelation?

**Response:** thank you very much for your valuable and logical inquiry. As we tried to make clear in the “data analysis and management” section of the manuscript we used two different types of regression analyses namely Logistic and Poisson for the first and second outcome variables, respectively. The space that let us use an independent t-test and ANOVA was the second outcome variable which is the receipt of the recommended number of BPCR messages which is a count variable. Most scholars recommended that to conduct such tests before we run a multivariable generalized linear model as a way of screening variables. So those two tests are simply part of the procedure we used to investigate the significant difference in the mean number of BPCR messages across categorical variables. Those variables that showed statistical significance were eligible for a generalized linear model (in this case, a multilevel mixed-effect negative binomial regression). The violation of the multivariate regression assumptions was checked prior to the start of the analysis, and we had no trouble doing so. Finally, please read the "Statistical methods and data analysis" section, Line 237-249, Page 8 of the "Revised manuscript with track changes."

**Comment 4:** Authors mentioned “Those variables with p-values less than 0.05 were eligible for a multilevel mixed-effect negative binomial regression using a generalized linear model (GLM) to determine the determinants of the number of BPCR messages received”. If they already exclude non-significant variables there is selection bias as they consider significant variables only. At least there should be inclusive criteria to include some variables with low p-value (significant and non-significant) using the rule of thumb or any selection criteria.

**Response:** thank you for your meticulous review and your insightful comment on the manuscript. In most studies and statistical literature level of significance in independent t-test and ANOVA were declared at p-value<0.05. We completely share your idea that this rule could be restrictive which might leads us to omit some important variables that significantly affect the uptake of recommended elements of BPCR messages. So, to avoid that we also checked for other important variables in previously conducted studies to assure their scientific plausibility. Fortunately, none of the important variables were omitted by the above tests and we proceeded to the next phase of multilevel analysis.

**Comment 5:** Under the model selection part: there first six lines are not about model selection. It is all about the random effect model and should be put under the statistical analysis part.

**Response:** thank you for your valuable comment and suggestion. As a result, we tried to clearly state the techniques we used to select the best models in the “Model building and selection” section of the "Revised manuscript with track changes.", Line 259-263, Page8

**Comment 6:** Model selection: the overdispersion might be by chance and the authors should indicate whether the variance is higher than the mean (using the rule of thumb or hypothesis testing)

**Response:** we appreciate your insightful comment and suggestion. Of course, It was also our concern prior to analysis that overdispersion can happen by chance. But as we read about it this might be rare in relatively larger sample sizes. As we read the analysis results, we noticed that the figures for the mean(1.25) and variance(2.51) had large disparities, making the occurrence of chance less likely. As a result, we performed the multi-level mixed effect negative binomial regression. Further, we will read about the way of conducting the rule of thumb and hypothesis testing.

**Comment 7:** It is strange that the outcome is described before the basic characteristics of the participants. describe sociodemographic characteristics before “The Overall uptake of WHO-recommended BPCR messages”

**Response:** Thank you so much for your thorough review and comment. We moved that section and placed it after we wrote all of the basic characteristics of study participants, as per your suggestion. After re-arrangement that specific section is placed in the “The Overall uptake of WHO-recommended BPCR messages” section of the "Revised manuscript with track changes.", Page 14

**Comment 8:** It is also recommendable that authors used a flow chart to show % of observations included in the final analysis (sample size >>excluded (reason)>>included into descriptive analysis>>included into outcome one (uptake)>>included for frequency of uptake etc.)

**Response:** We appreciate your valuable comment. We have reported this statement in narration form in the “The population of the study” section of the "Revised manuscript with track changes.", Line 134-139, Page5

**Comment 9:** Table one might be described in the graphs and narration of some results

**Response:** As per your suggestion we convert that table into a graph and now it is labeled as “Figure 1” in the revised version of the manuscript, Line 333-334, Page 14

**Comment 10:** For table 2, what type of estimation was used? describe how this estimation is done in the methodology. Is this estimation for all Ethiopian mothers? if not why didn't simply describe the summary statistics for the sample? Contraceptive utilization: what if contraceptive is used after pregnancy? Describe how contraceptive is measured and used in your data.

**Responses:** thank you very much for your insightful inquiry.

i. The estimations (test statistics in the form of ANOVA and independent t-test) were done to assess the significant difference in the mean number of BPCR messages across the covariates and it was mentioned in the “Statistical methods and data analysis” section "Revised manuscript with track changes.", Line 237-242, Page8.
ii. Contraceptive use, according to most studies, is a gateway to most maternal and child health services. If a mother used a contraceptive prior to her pregnancy, she is more likely to visit a health facility for prenatal care and has a better chance of receiving those BPCR messages.
iii. Regarding the variable measurement, we already mentioned the way of assessment of each variable in the “Measurement of variables of the study” section the under the sub-section “explanatory variables” during the initial submission. For more clarity, we have highlighted them in Lines 181-193, Page 6

**Comment 11:** Mixed model result: the base for most of the variables (region, religion, …) is not described in the method. So where is the variable selection described in the method part?

**Response:** We already have mentioned the way of variable selection in the “Statistical methods and data analysis” section of the "Revised manuscript with track changes.", Line 211-214, Page 7.

**Comment 12:** In table 5: the result of religion showed that protestant is increasing the odds of uptalking BPCR. This result is neither described in the result nor in the discussion. My question is what is the explanation for this result? did you check the adequacy of power and sample size to include all these variables?

**Response:** We greatly appreciate your meticulous review, which saved us from making such mistakes. During the analysis, none of the religions showed a significant association with the outcome variables. We typed 1.74 instead of 0.74 in the lower confidence interval and would like to apologize for the error, which we have corrected and highlighted in "Table 5".

**Comment 13:** Table 4: what is the denominator to calculate %? aren't we expecting total and respective freq. for each category?

**Response:** we agree with your suggestion and we compute the percentages with total and respective frequencies.

**Comment 14:** Enrollment in health insurance schemes decrease the odds of BPCR message uptake by 35%, so what will be the implication of this finding? What is the recommendation (will you recommend against the enrollment or any explanation for this outcome?)

**Response:** Thank you for your inquiry. The statement in the initial manuscript said that “Similarly, those respondents who didn’t enroll in a health insurance scheme were 35% less likely to receive BPCR messages as compared to their counterparts (AOR = 0.65; 95% CI: 0.42, 0.98)”. We kindly request that you re-read the statement for better clarity and is highlighted on Lines 374-376, Page 17,

**Comment 15:** Most of the reason you described is weak. Justify the discrepancies or associations in terms of scientific reasoning and theories do not guess (e.g., you justified the discrepancy of prevalence as the difference in sociodemographic characteristics of study participants, and health-system-related characteristics (like shortage of health care providers and distance to health facilities: aren't you included those factors in the model (already controlled)?

**Response:** We appreciate your feedback and suggestions, which will help us make the discussion section more relevant to the findings. As per your suggestion, we attempted to support the findings from various angles. The revised statements were highlighted in the “Discussion” section of the "Revised manuscript with track changes.", Line 425-433, Page 22.

**Comment 16:** did you include all the possible factors that could predict your outcome? If not, why don't you mention it under limitation?

**Response:** Thank you for your constructive feedback and suggestions. Following a thorough review of the literature, we attempted to incorporate all variables discovered to influence the uptake of maternal health services, particularly BPCR. As a result, we came to the consensus that this could not be our limitation, which is why we failed to mention it.

**__________________________________________________________________________________________________________END_______________________________________**

**THANK YOU!!!**

**Authors’ Response To Reviewer**#**2**

**Comment 1:** Table1: Number of BPCR messages received by women during their recent pregnancy preceding the survey in Ethiopia, 2016. Say something about the table since audiences/Readers did not understand the meaning.

**Response:** Thank you very much for taking the time to review our work and for your positive feedback. We received your thoughtful, and generous review, along with helpful feedback and suggestions, as a valuable contribution to our ongoing work. When it comes to elaboration, the current study has two outcome variables: receipt of at least one BPCR message (in a categorical) and receipt of the required number of BPCR messages. As stated in the method section, the reports in means and test statistics fall under the second outcome variable. The details were mentioned in the “Statistical methods and data analysis” section of the "Revised manuscript with track changes.", Line 226-239, Page 8.

**Comment 2:** Do not use abbreviations in the abstract part of the document.

**Response:** thank you for your suggestion and we have re-wrote those abbreviations in their full form throughout the abstract of the revised manuscript.

**Comment 3:** Describe EDHS 2016 Collected data under Ethical considerations.

**Response:** We appreciate your suggestion to incorporate some ethical issues regarding access and use of the data set. As per your suggestion, we have added some statements that were highlighted in the “Ethical consideration and consent to participate” section of the "Revised manuscript with track changes.", Line 265-268, Page 9.

**Comment 4:** The study relied on population-based, nationally representative data from 2016 Ethiopian Demographic and Health Survey (DHS), the fourth in a series of national-level population and health surveys carried out as part of the global Demographic and Health Survey (DHS) program. So, why only use EHDS 2016 Data for this study since the title on WHO Recommendations on BPCR messages?

**Response:** thank you for your insightful inquiry that we took it as our limitation and we might further try to see the trends in the uptake of BPCR messages across all the EDHS data.

**Comment 5:** this paper did not describe the population characteristics, Is there any exclusion data? not described, Did you check Completeness of Data?

**Response:** we appreciate your meticulous review. As per your suggestion, we have tried to revise the statements and the corrected versions were highlighted in the “The population of the study” section of the "Revised manuscript with track changes.", Line 134-139, Page 5.

**Comment 6:** under the limitation of the study, it says ''retrospective interviews of a selected group of women who had a live birth within five years of the survey''. Rewrite it.

**Response:** We have corrected the statement and the corrected form is highlighted in the “Discussion” section of the "Revised manuscript with track changes.", Line 512-514, Page 25.

**Comment 7:** What were/was drawback of EDHS2016 So far? you did not well explained.

**Response:** thank you for asking. We have mentioned the limitations of the study( EDHS) data in the last paragraph of the “Discussion” section. For more clarity we have highlighted them in Lines 512-518, page 25

**Comment 9:** Discussion should be rewrite it again.

**Response:** thank you for your suggestion that gives us the opportunity to revise the discussion section. Accordingly, we tried to revise and synthesize the entire section and the amendments were highlighted.

Finally, we appreciate your thorough review of our work, as well as your insightful remarks and suggestions.

__________________________________________________________________________________________________________END________________________________________

THANK YOU!!!
