# Supplementary material for: The uptake of WHO-recommended birth preparedness and complication readiness messages during pregnancy and its determinants among Ethiopian women: A multilevel mixed-effect analyses of 2016 demographic health survey
Source: PLoS One. 2023 Mar 23;18(3):e0282792. doi: 10.1371/journal.pone.0282792 (PMC10035894; doi:10.1371/journal.pone.0282792)
Supplement: Attachment — Submitted filename: Response to Reviewers.docx [file pone.0282792.s003.docx]

**AUTHORS’ RESPONSE TO ACADEMIC EDITOR AND REVIEWERS**

1. **Author’s Response To Academic Editor**

**Dear: Melkamu Merid Mengesha, MPH (Academic Editor)**

We thank you for a thorough reading and constructive comments and suggestions on our manuscript and for the opportunity to revise and resubmit. We are pleased to submit the revised research article “**The uptake of WHO-recommended birth preparedness and complication readiness messages during pregnancy and its determinants among Ethiopian women: A multilevel mixed-effect analyses of 2016 demographic health survey**” [PONE-D-22-24552R1] for your consideration in the special collection of Plos One. On the following pages, you will find our responses to the questions raised from your side. On behalf of my co-authors, I thank you for your consideration of this resubmission. We appreciate your time and look forward to your response.

Sincerely,

Aklilu Habte(MPH)(corresponding author)

[**aklilihabte57@gmail.com**](mailto:aklilihabte57@gmail.com)

**Journal requirement**

Please review your reference list to ensure that it is complete and correct. If you have cited papers that have been retracted, please include the rationale for doing so in the manuscript text, or remove these references and replace them with relevant current references. Any changes to the reference list should be mentioned in the rebuttal letter that accompanies your revised manuscript. If you need to cite a retracted article, indicate the article’s retracted status in the References list and also include a citation and full reference for the retraction notice.

**Response:** Thank you for providing us with the opportunity to revise our work by incorporating all of the valuable comments and suggestions made by the reviewers. We have checked all the references and we assured you that all of them were well-updated and non of them were retracted.

Additional Editor Comments

**Comment 1:** line 2-3: remove commas between texts in the title.

**Response:** thank you for your meticulous review, and we have corrected the statement by replacing the comma with the colon in the "title page" section of the “Revised manuscript with track changes”, Line 2, Page 1.

**Comment 2:** Lines 34-36: This statement starting in lines 34-36 in the abstract is not supported by evidence as the government of Ethiopia in partnership with international actors did a huge investment to improve maternal health.

**Response:** Thank you for your comment and suggestion, and we have corrected the statement and highlighted it in the "Abstract" section of the “Revised manuscript with track changes”, Line 34-35, Page 2.

**Comment 3:** expand abbreviations (CI and others in the document) on first use. use 'colon (:) instead of the 'semi-colon (;)'' to list the upper and lower confidence limits as in like [95% CI: X, Y].

**Response:** We appreciate your meticulous review and we already have written the expanded version for the abbreviation in the "Abstract" section of the “Revised manuscript with track changes”, Line 48, Page 2. The punctuation issue also has been corrected and highlighted in Line 50, page 2

**Comment 4:** Interpretation of results: present interpretations for individual and group level factors separately; do the same in the conclusion too. Interpretation of results should carefully reflect the nature of the design and model used.

**Response:** thank you for your vital comment that helps us to make the results of multi-level analyses clear to the readers by showing the nature of the design and model used. Accordingly, we added the individual and community-level factors that were significantly associated with the uptake of BPCR messages in the "Abstract" section of the “Revised manuscript with track changes”, Line 52-60, Page 2.

_____________________________END________________________________________

THANK YOU!!!
