# Supplementary material for: The uptake of WHO-recommended birth preparedness and complication readiness messages during pregnancy and its determinants among Ethiopian women: A multilevel mixed-effect analyses of 2016 demographic health survey
Source: PLoS One. 2023 Mar 23;18(3):e0282792. doi: 10.1371/journal.pone.0282792 (PMC10035894; doi:10.1371/journal.pone.0282792)
Supplement: Attachment — Submitted filename: Response to Reviewers.docx [file pone.0282792.s004.docx]

**AUTHORS’ RESPONSE TO ACADEMIC EDITOR AND REVIEWERS**

1. **Author’s Response To Academic Editor**

**Dear: Melkamu Merid Mengesha, MPH (Academic Editor)**

We thank you for a thorough reading and constructive comments and suggestions on our manuscript and for the opportunity to revise and resubmit. We are pleased to submit the revised research article “**The uptake of WHO-recommended birth preparedness and complication readiness messages during pregnancy and its determinants among Ethiopian women: A multilevel mixed-effect analyses of 2016 demographic health survey**” [PONE-D-22-24552R2] for your consideration in the special collection of Plos One. On the following pages, you will find our responses to the questions raised from your side. On behalf of my co-authors, I thank you for your consideration of this resubmission. We appreciate your time and look forward to your response.

Sincerely,

Aklilu Habte(MPH)(corresponding author)

[**aklilihabte57@gmail.com**](mailto:aklilihabte57@gmail.com)

**Journal requirement**

Please review your reference list to ensure that it is complete and correct. If you have cited papers that have been retracted, please include the rationale for doing so in the manuscript text, or remove these references and replace them with relevant current references. Any changes to the reference list should be mentioned in the rebuttal letter that accompanies your revised manuscript. If you need to cite a retracted article, indicate the article’s retracted status in the References list and also include a citation and full reference for the retraction notice.

**Response:** Thank you for providing us with the opportunity to revise our work by incorporating all of the valuable comments and suggestions made by the reviewers. We have checked all the references and we assured you that all of them were well-updated and non of them were retracted.

Additional Editor Comments

**Comment 1:** The length of the abstract is too much beyond the recommended 300 words count in the revised submission. Please check author PLOS ONE author guideline and revise this as a final opportunity to look into the abstract and other unnoticed issues (including editorials) throughout the document.

**Response:** Thank you for your thorough review; we have corrected the abstract section per the journal's requirements. In your previous comment, you suggested that individual and community level factors be reported in the abstract, and this was the sole reason that the abstract section was lengthened. Overall, we have attempted to reduce the lengthy abstract accordingly.

_____________________________END________________________________________

THANK YOU!!!
